# Functional characterization of *SDHB* variants: Advancing succinate dehydrogenase biology and variant curation in hereditary paraganglioma

**DOI:** 10.1172/JCI202727

**Published:** 2026-02-16

**Authors:** Roderick Clifton-Bligh

**Affiliations:** 1 Department of Endocrinology, Royal North Shore Hospital, Northern Sydney Local Health District, St Leonards, New South Wales, Australia.; 2 School of Medicine, Faculty of Medicine and Health, University of Sydney, Sydney, New South Wales, Australia.

## Abstract

Germline variants in the gene encoding succinate dehydrogenase subunit B (*SDHB*) occur in around 10% of all patients with pheochromocytomas and paragangliomas (PPGLs). Diagnosis of these variants has profound implications not only for the patient but also their first-degree relatives in terms of risk for PPGLs and other *SDHB*-associated tumors (renal cell cancer and gastrointestinal stromal tumors). Appropriate surveillance of *SDHB* variant carriers is associated with reduced mortality from these cancers. Curation of disease-causing (pathogenic) variants from benign variants is therefore crucial; however, this task is often difficult for missense variants when their impact on biological function is unclear. In this issue of the *JCI*, Lee et al. have described a newly developed cellular complementation assay for SDHB function that may assist variant curation in clinical practice and thereby improve outcomes for patients inheriting these cancer-risk variants.

## Introduction

Pheochromocytomas and paragangliomas (PPGLs) are highly heritable, and approximately 40% of cases are associated with familial syndromes involving heterozygous pathogenic mutations in at least 14 susceptibility genes (1). This strong genetic basis justifies near-universal genetic testing for patients with PPGL (2) but also creates a high burden of care for genetic testing laboratories to analyze and curate potential disease-causing variants. The clinical impact of such variant curation is emphasized further by inclusion of 8 PPGL susceptibility loci — *RET*, *VHL*, *MAX*, *TMEM127*, and the succinate dehydrogenase subunit and assembly factor genes *SDHB*, *SDHC*, *SDHD*, and *SDHAF2* — in a list of genes now considered essential to report even as secondary findings in exome/genome sequencing (3).

## *SDHx* variants

**Figure 1:**
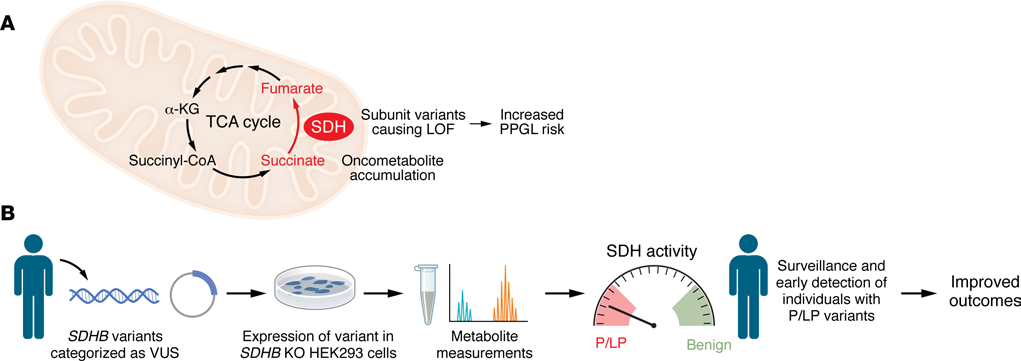
A high throughput assay to identify SDHB variants associated with SDH loss of function and PPGL risk. (**A**) The SDH enzyme converts succinate to fumarate in the Krebs (TCA) cycle, and variants in its subunits impair its function, leading to accumulation of succinate. Succinate is considered an oncometabolite due to its accumulation’s effects on α-ketoglutarate–dependent (α-KG-dependent) dioxygenases that are associated with tumor development. Pathogenic variants in SDH subunits, including in SDHB, are associated with developing PPGLs, renal cell cancers, and gastrointestinal stromal tumors. (**B**) Appropriate diagnosis and surveillance of SDHB carriers enables early detection of these tumors and improved outcomes. Curation of SDHB variants as pathogenic/likely pathogenic (P/LP) or benign can be difficult, and many variants remain classified as a variant of uncertain significance (VUS). Lee et al. developed an assay using transient transfection of SDHB-variant–containing plasmids into SDHB-KO HEK293 cells, followed by measuring succinate and fumarate by LC/MS-MS. The succinate/fumarate ratio is then transformed to a metric of SDH activity, which accurately distinguishes benign (high activity) from pathogenic variants (low activity).

The seminal recognition by Baysal et al. in 2000 (4) that *SDHD* was associated with familial paraganglioma quickly led to discovery of hereditary paraganglioma (PGL) syndromes associated with the other SDH subunit genes, among which *SDHB* (hereditary PGL syndrome 4, OMIM 185470) is the most frequent, occurring in around 10% of all PPGL cases, and more frequently associated with metastatic disease and poor survival (5, 6). *SDHx* are all classical tumor suppressor genes in Knudson’s 2-hit model wherein an inherited pathogenic variation in one allele in association with somatic loss of the other allele leads to complete SDH deficiency and tumorigenesis (7). *SDHx* pathogenic or likely pathogenic (P/LP) variants may be more common than initially suspected from the relative rarity of PPGL disease; inferred either from population allelic frequencies or from reportable genomic secondary findings, as many as 1 per 3,000 carry an *SDHx* P/LP variant (8, 9). Since appropriate surveillance of patients or family members with these variants has been shown in retrospective studies to be associated with reduced risk of metastatic disease and death (Figure 1A) (10, 11), there is an obligation for accurate diagnosis, starting with recognition and proper curation of gene variants. The American College of Medical Genetics and Genomics (ACMG) and the Association for Molecular Pathology (AMP) have defined standards for curating variants as P/LP or benign/likely benign (B/LB), using orthogonal approaches including frequencies of allelic variants in health versus disease, gene-disease specificity, allelic segregation with disease in families, and impact of allelic variants on gene function measured either directly in a validated assay or inferred from in silico tools (12). Unfortunately, many *SDHB* missense variants are currently unable to be classified into either P/LP or B/LB categories without determining their impact on protein function, and hence remain as variants of uncertain significance (VUS).

## SDH role in health and disease

SDH is chromosomally encoded but resides in mitochondria where it serves two roles: as Complex II in the electron transport chain of mitochondrial respiration, and catalyzing succinate to fumarate in the Krebs cycle (13). These 2 processes are intrinsically linked because optimum SDH enzymatic activity requires an electron acceptor (14). SDH deficiency leads to the Warburg effect of cells becoming reliant on glycolysis in place of mitochondrial ATP generation (15), and an accumulation of its substrate succinate inhibits several α-ketoglutarate–dependent dioxygenases such as hypoxia inducible factor prolyl dehydrogenase (HIF-PHD), TET demethylase, and histone demethylases (KDMs) (16). Since HIF accumulation and aberrant histone and DNA methylation are all traits of SDH-deficient tumors, succinate has become known as an oncometabolite (17).

## Assays for SDH deficiency

The most common assay for SDH deficiency in clinical use is immunohistochemistry (IHC) of tumors using antibodies against SDHB; in this context, loss of staining is highly sensitive and specific for pathogenic variants in any of the *SDHx* genes (18). Elevated succinate (or an elevated succinate/fumarate ratio) has also been used as a surrogate marker for SDH deficiency in tumors (19). Importantly, neither assay is allele specific; discovery of an *SDHx* genetic variant in a tumor with IHC-determined loss of SDHB or elevated succinate/fumarate does not prove that that variant is the cause of its SDH deficiency. Another shortcoming of these assays is the requirement for tumor tissue, which may be unavailable. Yeast assays have been used to assess allele-specific *SDHB* function but are cumbersome to set up and therefore are not scalable (20).

## A new, high-throughput assay for SDHB function

In this issue, Lee et al. (21) reported on the development of an allele-specific SDHB assay using *SDHB*-knockout HEK293 cells (SDHB-KO) and functional complementation by transient transfection of either WT or mutant *SDHB* constructs (Figure 1B). To facilitate high-throughput efficiency, their chosen read out was cellular content of succinate and fumarate measured by LC-MS/MS and expressed as a succinate/fumarate ratio; preliminary experiments had directly confirmed loss of SDH activity in SDHB-KO cells and restoration by transfection of wtSDHB by measuring complex II-specific oxygen consumption rates. To improve interassay standardization, the authors converted succinate/fumarate ratio into relative SDH activity by calibrating to mock- and wtSDHB-transfected SDHB-KO cells; this step was shown to improve diagnostic accuracy in later assessing *SDHB* VUS. The authors validated their assay using a reference set of variants with established clinical classifications; 9 B/LB variants restored estimated SDH activity to levels comparable with wtSDHB, whereas 27 P/LP variants failed to restore SDH activity to more than 11% of normal. The authors then applied their assay to resolve a collection of 34 *SDHB* VUS: 14 variants could be reclassified by functional impact as LP and 16 as LB. Their assay was found to have superior accuracy for identifying benign variants compared with the in silico tool REVEL. Notably, in one case from their own institution, an *SDHB* VUS identified as a secondary finding during clinical testing could be reclassified by their functional assay as LP and the patient was later found to have developed an abdominal PGL.

The authors extended their work to make 2 additional observations of immediate relevance to *SDHB* variant curation efforts: firstly, to show the critical importance of cysteine residues coordinating iron-sulfur clusters in SDHB; and secondly, prematurely truncating variants beyond codon 272 appeared to have no substantial effect on SDH activity (21). One final observation is intriguing but will require corroboration. Lee et al. suggested a graded genotype-phenotype relationship such that variants associated with near-complete loss of SDH activity are more likely to present with abdominal PPGLs; moderately impaired variants are more likely to present with head/neck PGLs; and mild hypomorphic variants are only associated with the recessive mitochondrial disorder known as Leigh syndrome.

This work generates many further questions, including whether variant impact on SDH activity is cell-type dependent, and whether some deleterious SDHB variants may preferentially affect electron transport or reactive oxygen species generation without abolishing succinate-to-fumarate catalysis. For ease of culture, Lee et al. used HEK293 cells for their assay, although they apparently found similar results in HeLa cells (21). It will be interesting to see whether functional impact of *SDHB* variants is replicated across many different cell lines, or perhaps SDH activity may be preserved for some of these variants in particular cell types. Naturally, it will be valuable to assess the utility of Lee et al.’s approach for other *SDH* subunit genes as well.

## Conclusion

This study will be of immediate translational relevance for *SDHB* variant curation, and, in turn, likely lead to important changes in clinical outcomes for patients with pathogenic *SDHB* variants at risk for often lethal paragangliomas.

## Version 1 02/16/2026

Electronic publication
